# Five-Year Remission of Hidradenitis Suppurativa Following the Removal of a Levonorgestrel Intrauterine Device and Disease Overview for Obstetrics and Gynecology Providers

**DOI:** 10.7759/cureus.28937

**Published:** 2022-09-08

**Authors:** Meredith H Hengy, Rachel Cevigney, Komalpreet Tur, Marlee Hewitt, Mary Ivey

**Affiliations:** 1 Department of Dermatology, Wayne State University School of Medicine, Detroit, USA; 2 Department of Obstetrics and Gynecology, Wayne State University School of Medicine, Detroit, USA; 3 Department of Ophthalmology, Wayne State University School of Medicine, Detroit, USA; 4 Department of Obstetrics and Gynecology, Bay View Obstetrics & Gynecology, Petoskey, USA

**Keywords:** gynecology, obstetrics, dermatology, intrauterine device, hidradenitis suppurativa

## Abstract

Hidradenitis suppurativa (HS) is a chronic inflammatory skin disease that is characterized by recurrent, painful nodules, sinus tract formation, and scarring. We report the case of a 20-year-old female with the abrupt onset of Hurley stage I HS following the implantation of a levonorgestrel intrauterine device (IUD) and complete remission of disease following the removal of the device one year later. To our knowledge, this is the first documented case of long-term remission of HS after the removal of levonorgestrel IUD. We also provide a concise overview of HS and suggested primary interventions for obstetrics and gynecology providers.

## Introduction

Hidradenitis suppurativa (HS) is a chronic inflammatory skin disease that is believed to be caused by follicular hyperkeratosis within the pilosebaceous-apocrine unit [1]. Hormonal influence is believed to play a prominent role in the pathophysiology of HS. It manifests clinically as tender, erythematous-to-hyperpigmented nodules, papules, and cysts localized to the intertriginous regions and is commonly associated with extensive scarring and sinus tract formation. There is often drainage of malodorous purulent material. HS is a chronic condition associated with disease flaring and varying severity, classified using the Hurley staging system (stages I-III) [2]. Diagnosis is primarily clinical with gram stain, culture, and sensitivity obtained when there is a concern for bacterial superinfection [2]. A biopsy is rarely needed to confirm the diagnosis. The treatment of HS is based on disease severity and options range from lifestyle changes to topical agents to systemic agents, including hormonal agents, oral antibiotics, and biologics. Surgical interventions and laser therapy are also used in some cases. Here, we present a case of HS related to the placement of a levonorgestrel intrauterine device (IUD).

## Case presentation

A 20-year-old female was referred to dermatology with a chief complaint of persistent nodules in the right inguinal skin fold and left axilla that began one month after the placement of a levonorgestrel IUD one year prior. The patient reported intermittent drainage of a purulent fluid from the right inguinal nodule. The patient had no personal or family history of these lesions prior to IUD placement. She was not taking any medications at the time. Her medical history was significant for mild acne vulgaris which was also worsened following the IUD placement. The patient was a non-smoker and within a normal body weight.

Examination of the right inguinal fold revealed a 1 cm firm, erythematous nodule with overlying tissue breakdown and drainage of a purulent material. Examination of the left axilla demonstrated a 1 cm erythematous nodule. There was no evidence of sinus tract formation or scarring around either lesion. A clinical diagnosis of Hurley stage I HS was established. This young woman was managed with an intralesional corticosteroid injection of both lesions and oral doxycycline and 10% benzoyl peroxide wash for five months, which provided temporary improvement in disease severity. However, the HS flared after the medication was discontinued. Other treatment options were discussed with the patient including a trial of clindamycin plus rifampin and spironolactone. The patient did not want to try any subsequent oral medications. Instead, due to the correlation of disease onset with IUD placement, the patient opted for IUD removal.

**Figure 1:**
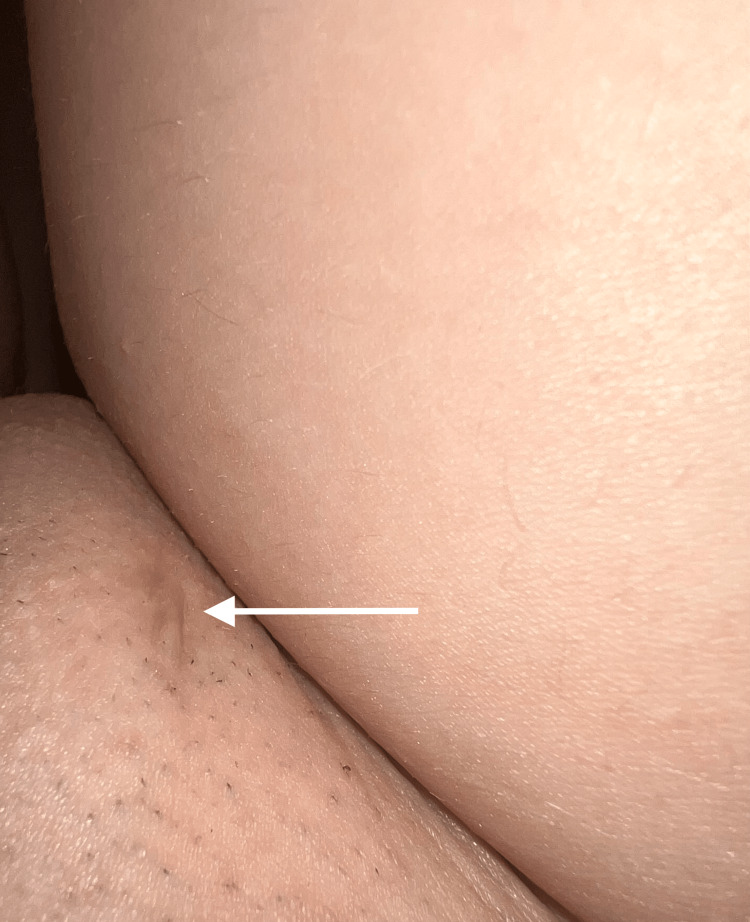
Scar on the lateral aspect of the right labia majora (white arrow).

Following the removal of the IUD, the skin healed gradually over a course of two months. The patient continued to follow up with dermatology and has not had any recurrence of HS in five years. She had a copper IUD placed one year after HS remission and has not experienced any HS flares. The patient has a persistent 1 cm scar on the lateral aspect of the right labia majora (Figure 1). There is no axillary scarring.

## Discussion

HS is a chronic inflammatory skin disease that is characterized by recurrent, painful nodules, abscesses, sinus tracts, and scars that commonly form in the axillary, peri-genital, anal, and inframammary regions of patients [1,2]. The diagnosis of the disease is largely based on patient history and clinical manifestation [2]. The severity of the disease is rated via Hurley staging, which is determined by the degree of abscess and sinus tract formation. Hurley stages I, II, and III correspond to mild disease, moderate disease, and severe disease, respectively (Table 1). Due to the intertriginous nature of this disease, obstetricians and gynecologists can reasonably be the first providers that detect HS presentations [3].

**Table 1:** Hidradenitis suppurativa staging and recommended interventions for obstetrics and gynecology providers.

Hurley stage	Description	Recommended intervention	Additional considerations
I	Single or multiple discrete abscesses. No sinus tract formation	10% benzoyl peroxide wash + topical clindamycin 1% lotion/solution daily	
II	Recurrent single or multiple abscesses. Limited sinus tract formation	10% benzoyl peroxide wash + topical clindamycin 1% lotion/solution daily PLUS Intralesional 10 mg/mL triamcinolone for acute flares PLUS 100 mg doxycycline daily for 1–6 months	Spironolactone 50 mg daily (titrate up to 200 mg daily) can be beneficial for patients who experience peri-menstrual flaring
III	Diffuse involvement of multiple interconnected sinus tracts and abscesses	10% benzoyl peroxide wash + topical clindamycin 1% lotion/solution daily PLUS 300 mg clindamycin twice daily + 300 mg rifampin twice daily for 8–10 weeks	Spironolactone 50 mg daily (titrate up to 200 mg daily) can be beneficial for patients who experience peri-menstrual flaring

The pathophysiology of HS is believed to be multifactorial, including genetic, mechanical, and dietary influences in conjunction with hormone imbalance [4]. The degree of hormonal influence on HS occurrence and progression has not been fully elucidated; however, HS occurs more frequently in female patients, especially of childbearing age, has a propensity to premenstrual flares, generally improves during pregnancy, and is often improved through antiandrogen therapy, suggesting a role of hormonal influence on the pathogenesis of HS [3,5-7]. In addition, various forms of hormonal birth control have been shown to affect HS, both positively and negatively. There are numerous hormonal birth control options, including oral contraceptives, patches, injections, vaginal rings, implants, and IUDs. These contraceptive options contain combinations of estrogen and progesterone or progesterone alone.

There is limited and anecdotal evidence suggesting that combined oral contraceptives (COCs) may worsen HS severity, particularly those with a higher progesterone-to-estrogen ratio. Alternatively, COCs with increased estrogen-to-progesterone ratios have been shown to improve HS symptoms and flares [8]. Worsening of HS has also been noted in women using medroxyprogesterone acetate and hormonal intrauterine devices, both of which are progesterone based [9]. North American Clinical Management Guidelines for HS were published in 2019, and antiandrogen contraceptives were a level II recommendation and a grade C recommendation [10].

The impact that hormonal contraceptives have on the severity of HS necessitates that prescribers remain cognizant when prescribing these medications to patients with a history of HS. In addition, we recommend that providers inquire whether patients are experiencing new skin findings that are concerning for HS after beginning hormonal medications, particularly those that contain a high progesterone-to-estrogen ratio or are progesterone only. A further suggestion is to consider contraceptives that contain the progestin, drospirenone, which is thought to act similarly to spironolactone, an antiandrogenic medication that has proven to help improve the severity of HS [11]. Non-hormonal birth control options, such as copper IUDs should also be considered. Lastly, it is important that providers are aware that the effect of some COCs may be reduced when patients are treated with rifampin, a commonly prescribed antibiotic for HS, due to the induction of the P450 enzyme [12].

Dermatologic treatment of HS is often based on disease severity. It is recommended that all patients with HS be referred to a dermatologist for thorough assessment and treatment of their disease. However, as obstetrics and gynecology providers may be the first to discover this disease, a good understanding of the various stages of HS, as well as first-line treatment strategies, is valuable (Table 1) [13].

To our knowledge, this is the first reported case in which long-term, complete remission of HS has been achieved after the removal of levonorgestrel IUD in a patient without a history of HS prior to IUD placement. Upon literature review, there are no other case reports of HS following IUD placement despite its known association with hormones. In this report, we provided a brief overview of HS and recommended a clinical management approach for obstetrics and gynecology providers.

## Conclusions

HS is a chronic inflammatory skin disease that has been associated with hormonal influences and is commonly encountered by obstetrics and gynecology providers. It is essential that these providers recognize the impact that hormonal changes can have on the development and progression of HS and tailor hormonal birth control options accordingly. Early recognition and intervention along with prompt referral to dermatology can help ease long-term disease burden.
